# Sociodemographic Characteristics and Dietary Habits Associated with Generalized Anxiety Disorder Symptoms in Adults Living in the Kingdom of Saudi Arabia: A Large Cross-Sectional Study

**DOI:** 10.3390/healthcare14162479

**Published:** 2026-08-11

**Authors:** Mai Adil Ghabashi

**Affiliations:** Department of Clinical Nutrition, Faculty of Applied Medical Sciences, Umm Al-Qura University, P.O. Box 715, Makkah 21955, Saudi Arabia; maghabashi@uqu.edu.sa

**Keywords:** generalized anxiety disorder, determinants, overweight, obesity, dietary habits, health status, Saudi Arabia

## Abstract

**Background:** Generalized anxiety symptoms represent an important public health concern in Saudi Arabia, yet evidence concerning their associated factors remains limited. Guided by the biopsychosocial framework, this study aimed to examine the independent associations of sociodemographic, health-related, psychological, and dietary factors with moderate-to-severe generalized anxiety symptoms among adults residing in Saudi Arabia. **Methods:** This cross-sectional study included an online convenience sample of 2044 adults. Participants completed self-reported sociodemographic, health-related, psychological, and dietary measures, together with the validated seven-item Generalized Anxiety Disorder scale (GAD-7) screening instrument. Multiple logistic regression was used to identify factors associated with moderate-to-severe generalized anxiety symptoms, defined as a GAD-7 score ≥ 10. **Results:** Overall, 33.2% of participants (n = 679) reported moderate-to-severe generalized anxiety symptoms. Women had higher odds of reporting these symptoms than men (odds ratio [OR] = 2.57, 95% confidence interval [CI]: 1.71–3.87; *p* < 0.001). Increasing age was associated with lower odds (OR = 0.97, 95% CI: 0.96–0.98; *p* < 0.001), whereas excess body weight was associated with higher odds (OR = 1.27, 95% CI: 1.02–1.59; *p* = 0.03). Daily consumption of soft drinks (OR = 1.62, 95% CI: 1.18–2.22; *p* = 0.002) and snacks (OR = 1.24, 95% CI: 1.00–1.54; *p* = 0.04) was also associated with higher odds. **Conclusions:** Within this predominantly female convenience sample, weight-related and dietary factors were associated with moderate-to-severe generalized anxiety symptoms. These findings may inform the development and evaluation of integrated mental health promotion initiatives, although prospective studies are needed to confirm the observed associations.

## 1. Introduction

The World Health Organization (WHO) defines mental health as a state of well-being where individuals recognize their potential, manage everyday stresses, and contribute positively to their communities [1]. However, this optimal state is interrupted by the fact that approximately 970 million individuals worldwide are affected by mental disorders, representing about one in eight people [2]. Globally, extensive research has highlighted the adverse impact of mental illness, particularly in terms of its health burden. According to the latest Lancet report, the global impact of mental illness is substantial, representing 32.4% of years lived with disability (YLDs) and 13.0% of disability-adjusted life years (DALYs) [3]. Anxiety disorders represent the most prevalent category of mental health disorders. The incidence of these disorders increased significantly from 31.13 million cases in 1990 to 45.82 million cases in 2019, marking a 47.19% rise over this period. Additionally, anxiety disorders accounted for 28.68 million cases of DALYs [4]. Several studies showed that high anxiety levels are associated with an increased probability of chronic diseases, including hypertension, diabetes, and cardiovascular disease [5,6,7].

In the Kingdom of Saudi Arabia (KSA), there is an increasing recognition of mental health challenges [8]. In particular, between 2025 and 2026, Saudi studies reported that approximately 33–35% of participants exhibited moderate-to-severe generalized anxiety symptoms [9,10]. Generalized anxiety disorder (GAD) is characterized by persistent and uncontrollable worry that adversely affects various aspects of life, including social, occupational, and personal functioning [11]. Therefore, the government aims to improve the quality of life for its citizens. This involves comprehensive reforms across various sectors, including healthcare, education, and social services, all aligned with the goals of the Saudi Vision 2030 framework. To address the burden of anxiety symptoms in the Saudi population, it is important to understand the factors associated with these symptoms.

In this context, the biopsychosocial framework [12] provides a useful perspective for examining anxiety disorders, as it highlights the complex interaction between biological, psychological, social, and behavioral determinants of health [13]. Consistent with this perspective, factors associated with anxiety disorders are multifaceted and may include a range of sociodemographic determinants such as age, gender, educational attainment, and socioeconomic status [14]. Cross-national data from 147,261 adults across 26 countries showed that GAD was more common among women, adults younger than 60 years, unmarried individuals, and those with lower educational attainment, lower household income, or unemployment [15]. In Saudi Arabia, moderate-to-severe GAD symptoms have been reported more frequently among women, younger adults, and students [16]. A nationwide screening study of 6015 adults also identified female sex, lower educational attainment, lower income, and smoking as factors associated with a positive GAD-7 screening result [17]. Psychological indicators are also central to the biopsychosocial model, with self-esteem, perceived quality of life, and health satisfaction representing relevant aspects of subjective well-being. Previous research has associated GAD with impaired quality of life and functioning [18], while a Saudi study found that higher self-esteem and health satisfaction were associated with lower odds of moderate-to-severe GAD symptoms [9].

Additionally, emerging research highlights the role of dietary habits in mental health, suggesting that nutrition can have a substantial impact on psychological well-being [19,20]. Nutrition-related evidence has demonstrated associations between anxiety symptoms and overall diet quality, micronutrient intake, and specific dietary exposures [21,22]. For example, a prospective study of more than 20,000 Australian women found that higher intakes of fruit, vegetables, fiber, and several other nutrient-rich foods were associated with a lower risk of incident anxiety, whereas higher processed meat and sodium intakes were associated with a higher risk after adjustment for age and socioeconomic status [20]. More recently, a 2026 systematic review and meta-analysis found that unhealthy dietary patterns were associated with higher odds of anxiety symptoms across adult and adolescent populations, although considerable heterogeneity across the included studies warrants cautious interpretation [23].

Among Saudi young adults, satisfactory nutrition knowledge was associated with lower GAD-7 scores and lower odds of moderate-to-severe symptoms [9], while another Saudi study found that higher GAD-7 scores were associated with poorer self-regulation of eating behavior [24]. Notably, several dietary behaviors commonly observed in Saudi Arabia deviate from dietary recommendations and have been associated with anxiety symptoms in studies conducted elsewhere. Inadequate fruit and vegetable intake is among the most pronounced dietary weaknesses in this population. According to a recent cross-sectional study conducted in 2025 examining the dietary habits of Saudi adults, it was reported that only 35% consumed fruits and vegetables on a daily basis. The daily consumption of snacks was substantially higher, reaching nearly 60%. In addition, about one-quarter of participants reported the daily intake of sugary foods and beverages, including soft drinks, and only around one-quarter consumed breakfast daily, with approximately three-quarters not consuming it on a regular basis [25]. These five components (daily intake of fruit, vegetables, breakfast, snacks and soft drinks) are of particular relevance to psychological well-being, as higher intake of fruits and vegetables has been linked to reduced psychological distress [26]. Similarly, daily breakfast consumption has been associated with lower odds of anxiety and depressive symptoms [27]. Conversely, frequent consumption of sugar-rich snacks and beverages has been associated with the severity of anxiety and depressive symptoms [28,29].

Existing Saudi research has largely focused on anxiety prevalence estimates or isolated correlates (e.g., sociodemographic correlates, nutrition knowledge, or eating self-regulation) rather than assessing relevant factors within a biopsychosocial framework. Examining sociodemographic and dietary factors alongside self-esteem, health satisfaction, and quality of life may enable a more comprehensive assessment of their independent associations with moderate-to-severe anxiety symptoms among this population. To the best of my knowledge, no previous large-sample Saudi study has jointly examined these factors within a biopsychosocial framework. The present study therefore aimed to determine their independent associations with moderate-to-severe generalized anxiety symptoms among adults residing in Saudi Arabia. Exploring these associations may generate useful insights to inform public health policies and mental health interventions, in line with the objectives of Saudi Vision 2030. The findings may also contribute to the existing literature and support the development of strategies to address mental health needs and promote population well-being.

## 2. Materials and Methods

### 2.1. Participants and Sample Characteristics

**Figure 1:**
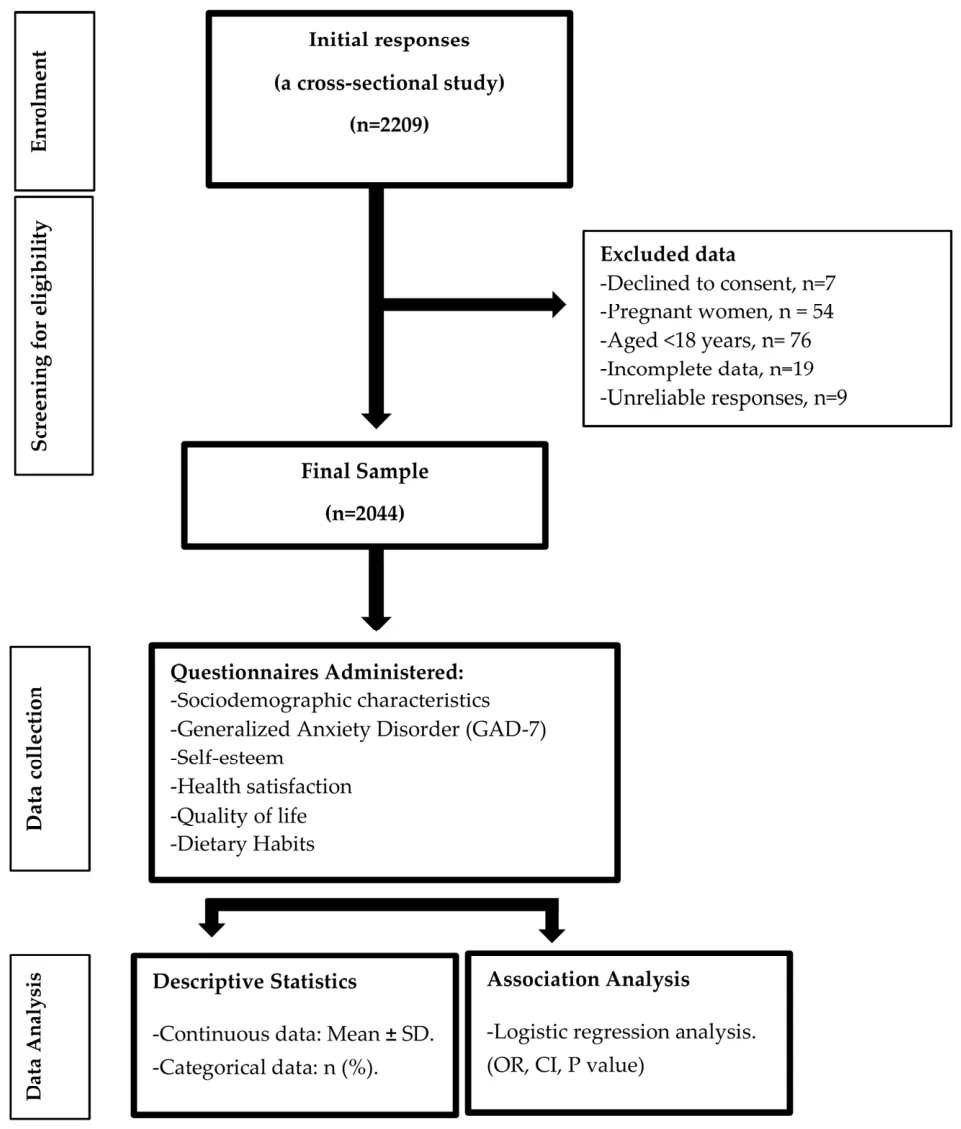
Overview of Participant Enrollment and Study Methodology. Abbreviations: CI, confidence interval; GAD-7, seven-item Generalized Anxiety Disorder scale; n, number of participants; OR, odds ratio; SD, standard deviation.

This cross-sectional study took place between December 2024 and June 2025. It was conducted via anonymous online surveys administered through Google Forms. Participants were recruited through an online survey distributed via social media platforms (i.e., WhatsApp, Telegram, and Snapchat), targeting adults residing in Saudi Arabia. A convenience sampling approach was used as the survey was distributed electronically. Eligibility criteria were as follows. Inclusion criteria were: (1) adults aged 18 years or older; (2) currently residing in Saudi Arabia; and (3) provision of electronic informed consent. Exclusion criteria were: (1) pregnancy, as it may influence body weight, dietary behavior, and psychological status; (2) Unreliable responses; and (3) questionnaires with missing GAD-7 outcome data were excluded. Formal psychiatric diagnoses were not assessed; therefore, participants were not excluded based on psychiatric comorbidity, and these comorbidities could not be controlled for in the analyses. This approach was consistent with the study’s aim of assessing generalized anxiety symptoms in a nonclinical adult sample rather than clinically diagnosed GAD. The final sample size comprised 2044 participants. The flow diagram illustrating participant selection and the research methodology is presented in Figure 1.

With regard to ethical considerations, this study was conducted in accordance with the principles of the Declaration of Helsinki and was approved by the Institutional Ethics Committee of Umm Al-Qura University (Approval No. HAPO-02-K-012-2024-12-2387). Participation was voluntary, and electronic informed consent was obtained from all participants before completing the questionnaire. No personally identifiable information was recorded. All responses were stored securely and analyzed in aggregated form to ensure participant confidentiality. Participants did not receive financial compensation for their participation.

Data analysis included 2044 participants residing in the KSA, with a mean age of 28.74 ± 12.84 years. The majority of participants were Saudi Arabian (92%; n = 1893). Most of them were healthy (78.5%; n = 1605), while 21.5% (n = 439) were diagnosed with a medical condition. The prevalence of overweight and obesity was high, with 23.3% (n = 476) classified as overweight and 19.8% (n = 405) classified as obese (Table 1). A substantial portion of the participants, reaching one-third of the sample (33.2%; n = 679), reported moderate or severe generalized anxiety symptoms. Approximately one in three participants had GAD-7 scores in the moderate-to-severe range (Table 2). The intake of fruits and vegetables among the participants was remarkably low. Specifically, only 11.9% reported consuming fruits daily (n = 244), while 23.5% reported daily vegetable consumption (n = 481). In contrast, over a quarter of the participants (28.1%) reported daily consumption of snacks (n = 575). Furthermore, about 10% of these participants indicated that they consumed soft drinks daily (n = 206; Table 1).

### 2.2. Conceptual Framework and Variable Selection

The selection of variables included in this study was guided by the biopsychosocial framework of mental health, which proposes that anxiety disorders arise from interactions between sociodemographic, lifestyle/behavioral, and psychological factors [30]. Accordingly, the study included key sociodemographic variables (age and gender), health-related indicators (body mass index [BMI] and disease status), psychological indicators (self-esteem, health satisfaction, and quality of life), and dietary habits (daily intake of vegetables, fruits, snacks, soft drinks, and breakfast). Rather than conducting an exhaustive dietary assessment, these five food components were purposively selected because they reflect well-documented dietary gaps in the Saudi adult population and have established associations with physical and mental health outcomes, thereby representing the greatest public-health relevance.

### 2.3. Study Tools and Data Collection

The data collection process included the following sociodemographic variables: age, gender, nationality, marital status, and educational status. In addition, health status and self-reported measurements of height and weight were collected. Self-reported height and weight enabled anthropometric data to be collected from a large, geographically dispersed online sample for whom direct measurement was not feasible. Body mass index (BMI) was calculated by dividing the weight in kilograms by the square of height in meters. Based on the computed BMI values, participants were classified into established weight categories: underweight (BMI < 18.5 kg/m^2^), normal weight (BMI 18.5–24.9 kg/m^2^), overweight (BMI 25.0–29.9 kg/m^2^), and obese (BMI ≥ 30.0 kg/m^2^). In addition to these metrics, the study also collected data on various dimensions of psychological well-being. Further details are presented below.

#### 2.3.1. Generalized Anxiety Disorder (GAD) Symptoms

GAD symptoms were evaluated using a validated 7-item scale (GAD-7) [31]. It is a widely used screening instrument for assessing the severity of generalized anxiety symptoms, with well-established psychometric properties. The Arabic version of the scale has demonstrated good reliability and validity in the Saudi population, with acceptable internal consistency (Cronbach’s α = 0.763) as indicated by AlHadi et al. [32]. In the present study, the GAD-7 showed good internal consistency (Cronbach’s α = 0.857), supporting the reliability of the GAD-7 scores in this sample. Additionally, the Saudi Arabian Ministry of Health has endorsed the Arabic version of this questionnaire as an initial anxiety screening tool. The GAD-7 items focus on various symptoms of GAD, with each question rated on a 4-point Likert scale ranging from 0 (not at all) to 3 (nearly every day). Scores can range from 0 to 21, with higher totals indicating greater levels of anxiety. Anxiety severity is categorized as follows: 0–4 for minimal anxiety, 5–9 for mild anxiety, 10–14 for moderate anxiety, and 15–21 for severe anxiety. Accordingly, the cutoff score of ≥10 was used to separate moderate-to-severe anxiety symptoms from minimal to mild anxiety symptoms, where scores below 10 indicate the latter [33]. However, it is important to note that this cutoff does not establish a clinical diagnosis of GAD.

#### 2.3.2. Self-Esteem

Self-esteem was measured using the validated Arabic version of the single-item self-esteem scale [34], which asked participants to evaluate the statement, “I have high self-esteem.” This scale employed a 5-point Likert format, where responses ranged from “not at all true of me” to “very true of me.” The scoring system was as follows: 1 = not at all true, 2 = somewhat untrue, 3 = somewhat true, 4 = mostly true, and 5 = very true. Participants chose the option that best represented their level of agreement with the statement. A higher score indicated greater self-esteem, while a lower score suggested lower self-esteem. Responses were categorized into two groups: ‘Low self-esteem’ (including scores of 1 to 3) and ‘High self-esteem’ (including scores of 4 and 5).

#### 2.3.3. Health Satisfaction

Health satisfaction was evaluated by asking participants, “How satisfied are you with your health?” This question was adapted from the validated World Health Organization Quality of Life-Brief Version (WHOQOL-BREF) questionnaire [35]. Respondents were given a 5-point Likert scale with the following response options: (1) very dissatisfied, (2) dissatisfied, (3) neither dissatisfied nor satisfied, (4) satisfied, and (5) very satisfied. Responses were divided into two groups: ‘Low health satisfaction’ (comprising responses 1 to 3) and ‘High health satisfaction’ (including responses 4 and 5).

#### 2.3.4. Quality of Life

Quality of life was assessed by asking participants, “How would you rate your quality of life?” This question was adapted from the Arabic version of the WHOQOL-BREF [35]. Respondents were given a 5-point Likert scale with the following response options: (1) very poor, (2) poor, (3) neither poor nor good, (4) good, and (5) very good. Responses were divided into two groups: ‘Low quality of life’ (comprising responses 1 to 3) and ‘High quality of life’ (including responses 4 and 5).

#### 2.3.5. Dietary Habits

Dietary habits were assessed using five items selected from a previously published 20-item food-frequency questionnaire. This questionnaire is a brief tool designed to measure food intake in Arabic-speaking populations [36]. These five items were purposively selected, including daily intake of vegetables, fruits, snacks, soft drinks, and breakfast, because these components represent well-documented deficiencies in the Saudi adult diet, each with an established link to physical or mental health. Together, they were included as selected indicators of dietary behavior within the lifestyle domain of the biopsychosocial framework, rather than as a comprehensive assessment of dietary intake. For each item, participants indicated their consumption frequency, selecting from options ranging from “never” to “once or more daily.” Responses were subsequently dichotomized into daily consumption versus less-than-daily consumption. This binary classification was adopted for several reasons. First, for the core foods of interest, daily intake represents the clinically and behaviorally meaningful threshold: national and international dietary guidance frames fruit and vegetable recommendations in terms of daily servings, such that daily versus non-daily consumption directly reflects whether a protective habit is established. Likewise, for soft drinks and snacks, daily consumption denotes habitual rather than occasional intake and is the pattern most strongly associated with adverse metabolic and psychological outcomes, whereas infrequent consumption is unlikely to carry comparable risk. Second, dichotomization at the daily threshold reduced sparse-cell counts across the intermediate frequency categories, thereby improving the stability of the logistic regression estimates and avoiding unreliable odds ratios that can arise from thinly populated ordinal categories. Third, a consistent binary coding scheme was applied across all behavioral and psychological variables in the study (see Section 2.3.2, Section 2.3.3 and Section 2.3.4), ensuring interpretive uniformity across predictors. This approach yields readily interpretable odds ratios contrasting individuals with an established daily habit against all others, which is directly aligned with the study’s public-health aim of identifying modifiable dietary behaviors.

### 2.4. Procedure

After accessing the online survey, participants reviewed the study information and provided electronic informed consent. They then completed the questionnaire sections in a fixed order: sociodemographic and anthropometric information, including age, gender, marital status, height, and weight; GAD-7; self-esteem, health satisfaction, and quality of life; and, finally, dietary habits. Responses were collected anonymously through Google Forms. Before analysis, the data were screened for eligibility, completeness, and unreliable entries.

### 2.5. Statistical Analysis

Data analysis was conducted using IBM Statistical Package for the Social Sciences (SPSS) Statistics, version 28.0 (IBM Corp., Armonk, NY, USA). The normality of the data was assessed through scatter plots and histograms. Skewness values for the numerical variables ranged from −0.653 to 1.217, while kurtosis values ranged from −0.496 to 0.553. All values were within the acceptable range of −2 to +2, indicating no substantial univariate distributional abnormalities. Continuous variables were reported as mean ± standard deviation, while categorical variables were expressed as frequency (%). Multiple logistic regression analysis was used to investigate factors associated with moderate-to-severe generalized anxiety symptoms. Before regression analysis, the GAD-7 total score was dichotomized into moderate-to-severe symptoms (≥10, coded 1) and minimal-to-mild symptoms (<10, coded 0). Age was entered as a continuous variable. The remaining predictors were entered as binary contrasts: female versus male, Saudi versus non-Saudi, disease versus healthy status, overweight/obesity versus underweight/normal weight, and university versus non-university education. Self-esteem, quality of life, and health satisfaction were categorized as high (scores 4–5) versus low (scores 1–3), while dietary variables were categorized as daily versus less-than-daily intake. The second category in each contrast served as the reference group. Consistent with the study objective, this coding was selected to provide interpretable comparisons between substantively defined participant groups regarding their odds of screening positive for moderate-to-severe generalized anxiety symptoms, although categorization may reduce granularity and information compared with retaining the variables in their original forms. The odds ratio (OR) was calculated to estimate the odds of moderate-to-severe generalized anxiety symptoms in relation to the included variables. A 95% confidence interval (CI) was determined for the estimated coefficients, which indicates the range in which the true population parameter is expected to lie with 95% confidence. A significance level of *p* < 0.05 was established.

The required sample size for logistic regression analysis was estimated using the rule of at least 10 outcome events per predictor variable. Considering the 14 predictor parameters included in the model, a minimum of 140 outcome events was required. The final sample of 2044 participants yielded substantially more outcome events than this minimum and was therefore considered adequate for the planned logistic regression analysis. Multicollinearity among predictor variables was assessed using the variance inflation factor (VIF). All VIF values were below 2 (ranging from 1.05 to 1.61), indicating no evidence of problematic multicollinearity. Model fit was evaluated using the Hosmer–Lemeshow goodness-of-fit test, and the explanatory power of the model was assessed using the Nagelkerke R^2^ statistic. Given the predominance of female participants in the sample, a sensitivity analysis restricted to female respondents was conducted to assess the robustness of the observed associations. The results remained largely consistent with those of the main model.

## 3. Results

### Logistic Regression Analyses of Factors Associated with Moderate-to-Severe Generalized Anxiety Symptoms

As shown in Table 3, the results of the logistic regression analysis provided insight into the factors associated with moderate-to-severe generalized anxiety symptoms in the population living in the KSA. The findings indicated that age was significantly associated with the reported moderate-to-severe symptoms. Older individuals had lower odds of reporting moderate-to-severe symptoms compared to younger people. Specifically, each additional year of age was associated with a 3% decrease in the odds of reporting moderate-to-severe anxiety symptoms (OR: 0.97, 95% CI: 0.96 to 0.98, *p* < 0.001). In addition to age, the analysis revealed sex differences in the odds of moderate-to-severe generalized anxiety symptoms. Females had more than twice the odds of reporting moderate-to-severe generalized anxiety symptoms compared with males (OR: 2.57, 95% CI: 1.71 to 3.87, *p* < 0.001). Notably, being overweight or obese was associated with 27% higher odds of moderate-to-severe generalized anxiety symptoms (OR: 1.27, 95% CI: 1.02 to 1.59, *p* = 0.03). Moreover, individuals suffering from health problems were found to have 64% higher odds of reporting moderate-to-severe generalized anxiety symptoms (OR: 1.64, 95% CI: 1.27 to 2.11, *p* < 0.001).

Conversely, all three psychological indicators were inversely associated with the outcome. High self-esteem, high quality of life, and high health satisfaction were associated with 31%, 32%, and 40% lower odds of reporting moderate-to-severe generalized anxiety symptoms, respectively (self-esteem: OR: 0.69, 95% CI: 0.56 to 0.86, *p* < 0.001; quality of life: OR: 0.68, 95% CI: 0.55 to 0.85, *p* < 0.001; health satisfaction: OR: 0.60, 95% CI: 0.48 to 0.74, *p* < 0.001). Lastly, the study investigated the association between dietary habits and generalized anxiety symptoms. The findings revealed that the daily intake of soft drinks was associated with 62% higher odds of reporting moderate-to-severe generalized anxiety symptoms (OR: 1.62, 95% CI: 1.18 to 2.22, *p* = 0.002). Similarly, the daily intake of snacks was found to be associated with 24% higher odds of reporting moderate-to-severe generalized anxiety symptoms (OR: 1.24, 95% CI: 1.00 to 1.54, *p* = 0.04). The sensitivity analysis restricted to female participants yielded results largely consistent with those of the main model (Table 4).

## 4. Discussion

The present study investigated the proportion of participants reporting moderate-to-severe generalized anxiety symptoms and its associated factors in the KSA. The findings indicated that a substantial proportion of participants reported moderate-to-severe generalized anxiety symptoms. Age and certain dietary habits showed significant associations with the odds of moderate-to-severe generalized anxiety symptoms. Approximately one-third of participants reported moderate-to-severe generalized anxiety symptoms. These findings are consistent with recent Saudi studies published between 2025 and 2026 reporting that roughly 33–35% of participants had moderate-to-severe generalized anxiety symptoms [9,10]. However, direct comparison with the 12.3% 12-month prevalence estimate reported by the Saudi National Mental Health Survey should be made cautiously because of differences in sampling strategies, assessment methods, and outcome definitions [37]. This underscores the urgent need to identify the factors associated with moderate-to-severe generalized anxiety symptoms in order to inform the development of targeted interventions that can effectively address this issue.

The findings of the present study indicate that certain sociodemographic factors were significantly associated with generalized anxiety symptoms. For instance, older adults had lower odds of reporting moderate-to-severe symptoms than their younger counterparts. This association may reflect age-related differences in life experience or coping strategies [38]. Additionally, females had more than twice the odds of reporting moderate-to-severe symptoms compared to males. This aligns with previous research indicating a higher incidence of anxiety disorders among women, which may be linked to biological, hormonal, and sociocultural factors [39]. However, the cross-sectional design does not permit determination of the temporal direction or mechanisms underlying these associations. Nevertheless, these findings may help inform consideration of demographic differences in future mental health research and public health planning, particularly regarding younger adults and women in the KSA.

Notably, the present study demonstrated that, alongside sociodemographic factors, unhealthy dietary habits including the daily consumption of snacks (such as cake, biscuits, and chips) and soft drinks were associated with higher odds of moderate-to-severe symptoms within this population. These findings are consistent with the existing literature [40,41,42]. One possible explanation for the observed association is the high sugar content of these foods, although the present study cannot establish this mechanism. Consistent with this possibility, systematic reviews have reported a potential positive association between the intake of highly sugary foods and anxiety disorders [28] as well as depression [29]. An additional recent umbrella review encompassing eight systematic reviews has also reported associations between unhealthy dietary components, particularly sugary items, and poor mental health and well-being [43]. Conversely, replacing unhealthy dietary habits with healthier alternatives may contribute to an improvement in psychological well-being. For instance, a prior short-term intervention study found positive effects of replacing unhealthy snacks, such as chocolate and chips, with healthier alternatives such as fruits, on mental health outcomes. The study engaged 100 college student participants over a 10-day period, revealing that those who consumed fruits demonstrated a notable reduction in anxiety levels compared to participants who consumed unhealthy snacks [44]. However, such potential mechanisms were not examined in the present study and cannot be inferred from the cross-sectional findings.

Overall, higher fruit and vegetable intake has been associated with better mental well-being. Evidence from a systematic review of 61 studies suggests that a higher intake of these foods is correlated with increased optimism and reduced psychological distress [26]. Moreover, it was found that individuals who consumed fruits daily were three times more likely to exhibit effective self-regulation in their eating behaviors, a factor that has been associated with lower odds of obesity as well as generalized anxiety symptoms [24]. Unfortunately, the findings of the current study indicate that daily consumption of fruits and vegetables is notably low, with only 11.9% of participants consuming fruits and 23.5% consuming vegetables on a daily basis. This underscores the necessity for effective strategies to promote adequate intake of these food groups within the Saudi population.

The current study also indicated that overweight and obesity were found to be associated with higher odds of moderate-to-severe generalized anxiety symptoms. This is consistent with the existing literature [45]. Several biological mechanisms may help explain the associations observed between dietary behaviors, body weight, and anxiety symptoms. Previous research suggests that anxiety disorders are linked to dysregulation of the hypothalamic–pituitary–adrenal (HPA) axis, a central component of the body’s stress response system [46]. Persistent activation of this pathway may alter cortisol regulation and increase vulnerability to anxiety symptoms [47]. In addition, inflammatory processes have been proposed as another potential mechanism, as elevated levels of pro-inflammatory cytokines have been associated with both obesity and anxiety-related conditions [48]. Dietary behaviors may further interact with these biological pathways, since frequent consumption of ultra-processed foods and sugar-sweetened beverages has been linked to increased systemic inflammation and poorer mental health outcomes [49]. Nevertheless, because the present study was cross-sectional, temporality cannot be established. Reverse causation is possible [50]: individuals with greater anxiety symptoms may consume snacks or soft drinks more frequently or experience weight changes through emotional eating or other less healthy dietary habits. Residual confounding by unmeasured factors such as sleep quality, physical activity, social class and diagnosed psychiatric comorbidities may also partly explain the observed associations. Such potential confounding influences on the observed associations cannot be ruled out, and further investigations should measure and control for these factors. Additionally, individuals struggling with overweight or obesity may experience social stigma, body dissatisfaction, and lower self-esteem, which may also be relevant to the observed association between body weight and anxiety symptoms [51].

Although causal effects cannot be inferred from the present findings, interventions promoting healthy weight management and healthier dietary habits may warrant evaluation as potential components of mental health promotion in the KSA. Prospective and intervention studies are needed to determine the temporal direction of the observed associations and to examine whether modifying dietary behaviors or body weight influences anxiety symptoms. Digital interventions may support healthier dietary behaviors, although their effectiveness varies across settings and intervention designs [52]. Digital technologies can improve the accessibility and flexibility of healthcare delivery [53]. In support of their application to nutrition education, a digital nutrition education intervention improved nutrition knowledge among information technology professionals [54]. A systematic review of 27 studies identified several features associated with successful online nutrition education interventions delivered through websites, smartphone applications, online courses, and text messaging [55]. However, evidence on the effectiveness of digital health interventions in the KSA remains limited [56,57]. This limited evidence makes it difficult to identify the enablers and barriers to participation in these programs among the Saudi population. Therefore, it is important to investigate what motivates and enables young adults in the KSA to engage with digital nutrition education. Understanding these cultural and contextual factors could help develop effective, tailored interventions that improve the overall health and well-being of the Saudi community.

### Strengths and Limitations

This study has several strengths. First, it included a relatively large sample of adults residing in Saudi Arabia, which enhances the statistical power of the analyses and allows the examination of multiple factors associated with anxiety symptoms. Second, the study incorporated variables from multiple domains—including sociodemographic characteristics, lifestyle behaviors, and psychological indicators—guided by the biopsychosocial framework of mental health. This approach provides a better understanding of factors associated with generalized anxiety symptoms. Third, anxiety symptoms were assessed using the well-established Generalized Anxiety Disorder scale (GAD-7), a widely validated screening instrument that has demonstrated good psychometric properties in both international and Arabic-speaking populations.

However, several limitations should be acknowledged. First, the cross-sectional design of the study does not allow conclusions regarding causal relationships, and therefore the findings should be interpreted as associations rather than causal effects. Second, although the questionnaire was brief, the fixed sequence of measures may have introduced context or priming effects, and the absence of counterbalanced administration prevented assessment of potential questionnaire-order effects. Third, women constituted 90.5% of the study sample. This marked sex imbalance may have influenced the observed frequency of moderate-to-severe anxiety symptoms and limits the generalizability of the findings, particularly to male participants; therefore, findings concerning men should be interpreted cautiously. Fourth, the study relied on online convenience sampling, which may have introduced selection bias, as participation was more likely among individuals who were younger, female, more active on social media, or more interested in health-related topics. Adults younger than 30 years constituted 66.4% of the sample, indicating that younger adults were overrepresented. Consequently, the observed frequency of moderate-to-severe anxiety symptoms should be considered specific to the study sample and should not be interpreted as a population-level prevalence estimate. In addition, the regional distribution of participants across Saudi provinces was not examined. Future studies should use region-based sampling and recruit samples with more balanced representation of women and men, ensuring adequate male participation to support reliable sex-stratified analyses.

Fifth, an additional limitation relates to the assessment of dietary habits. The dietary variables were derived from a brief food frequency questionnaire and included a limited number of food categories. While these items provide useful indicators of general dietary behaviors, they do not capture the full complexity of dietary patterns or total nutrient intake. Additionally, several variables, including height and weight, were self-reported, which may introduce measurement bias. Finally, although socioeconomic variables such as income and employment status may influence anxiety outcomes, these factors were not included in the present survey in order to maintain a concise questionnaire and minimize respondent burden in the online format. In addition, limiting the number of variables helped maintain the statistical stability of the regression model. Consistent with this, the regression models showed modest explanatory power (Nagelkerke R^2^ = 0.132–0.138), a value that is common for logistic models of binary psychosocial outcomes and reflects the multifactorial nature of anxiety rather than model misspecification. Model calibration was nonetheless supported by a non-significant Hosmer–Lemeshow test. These findings indicate that the measured factors capture only part of the risk, and that additional unmeasured determinants such as sleep quality, genetic predisposition, stressful life events, and broader socioeconomic conditions may have influenced the observed findings. Accordingly, the results should be interpreted as identifying modifiable correlates of generalized anxiety symptoms rather than as a comprehensive explanatory model.

## 5. Conclusions

This study examined sociodemographic, psychological, health-related, and dietary characteristics associated with moderate-to-severe generalized anxiety symptoms within a large online convenience sample of adults residing in Saudi Arabia. Within this study sample, 33.2% of participants screened positive for moderate-to-severe anxiety symptoms; however, this sample-specific proportion should not be interpreted as a population-level prevalence estimate. Female sex and younger age were associated with higher odds of moderate-to-severe anxiety symptoms. Overweight or obesity and the daily consumption of soft drinks and snacks were also associated with higher odds of these symptoms. These findings identify potentially relevant demographic and lifestyle correlates that warrant further investigation. However, because women and adults younger than 30 years were overrepresented, the findings should not be generalized to the broader Saudi adult population. Future studies using probability-based or region-stratified sampling, with more balanced representation across sex and age groups, are needed to confirm these associations and inform appropriately targeted mental health promotion and healthy lifestyle initiatives.

## Figures and Tables

**Table 1:** Sociodemographic characteristics and dietary habits of the study participants.

Variable	Participants (n = 2044)	
**Age, years**	28.74	±12.84
(<30 y)	1357	(66.4)
(≥30 y)	687	(33.6)
**Sex, n (%)**		
Male	194	(9.5)
Female	1850	(90.5)
**Nationality, n (%)**		
Saudi	1893	(92.6)
Non-Saudi	151	(7.4)
**Educational status, n (%)**		
With university degree	1241	(60.7)
Without university degree	803	(39.3)
**BMI, kg/m^2^**	24.76	±5.96
**Body weight, n (%)**		
Underweight	294	(14.4)
Normal	869	(42.5)
Overweight	476	(23.3)
Obesity	405	(19.8)
**Health status**		
Healthy	1605	(78.5)
Diagnosed with a disease	439	(21.5)
**Dietary habits**		
Daily intake of vegetables		
No	1563	(76.5)
Yes	481	(23.5)
Daily intake of fruits		
No	1800	(88.1)
Yes	244	(11.9)
Daily intake of breakfast		
No	1132	(55.4)
Yes	912	(44.6)
Daily intake of snacks		
No	1469	(71.9)
Yes	575	(28.1)
Daily intake of soft drinks ^a^		
No	1837	(89.9)
Yes	206	(10.1)

Continuous variables are presented as mean ± SD; categorical variables are presented as n (%). ^a^ One response was missing for daily soft-drink intake; percentages were calculated using available cases (n = 2043). Abbreviations: BMI, body mass index; n, number of participants; SD, standard deviation; y, years.

**Table 2:** Psychological characteristics of study participants.

Variable	Overall (n = 2044)	
**GAD-7 score**	8.13	±4.64
**GAD-7 symptom severity, n (%)**		
Minimal/Mild	1365	(66.8)
Moderate/Severe	679	(33.2)
**Self-esteem score**	3.89	±0.96
**Level of self-esteem, n (%)**		
High	1368	(66.9)
Low	676	(33.1)
**Quality of Life score**	3.83	±0.87
**Level of Quality of Life, n (%)**		
High	1351	(66.1)
Low	693	(33.9)
**Health satisfaction score**	3.45	±1.06
**Level of health satisfaction, n (%)**		
High	1070	(52.3)
Low	974	(47.7)

Continuous variables are presented as mean ± SD; categorical variables are presented as n (%). Abbreviations: GAD-7, seven-item Generalized Anxiety Disorder scale; n, number of participants; SD, standard deviation.

**Table 3:** Factors Associated with Moderate-to-Severe Generalized Anxiety Symptoms: Logistic Regression Analysis.

Variables	OR	(95% CI)	*p* Value
Age, years	0.97	(0.96, 0.98)	<0.001
Gender, female	2.57	(1.71, 3.87)	<0.001
Nationality, Saudi	1.02	(0.70, 1.48)	0.9
Health status, disease existence	1.64	(1.27, 2.11)	<0.001
Weight status, overweight and/or obesity	1.27	(1.02, 1.59)	0.03
Education, with a university degree	0.87	(0.70, 1.10)	0.2
High self-esteem	0.69	(0.56, 0.86)	<0.001
High quality of life	0.68	(0.55, 0.85)	<0.001
High health satisfaction	0.60	(0.48, 0.74)	<0.001
Daily intake of vegetables	0.89	(0.68, 1.15)	0.3
Daily intake of fruits	1.00	(0.71, 1.41)	0.9
Daily intake of snacks	1.24	(1.00, 1.54)	0.04
Daily intake of soft drinks	1.62	(1.18, 2.22)	0.002
Daily intake of breakfast	0.89	(0.72, 1.09)	0.2

Abbreviations: CI, confidence interval; n, number of complete cases; OR, odds ratio. The analysis was based on complete cases (n = 2043).

**Table 4:** Sensitivity Analysis Restricted to Female Participants: Logistic Regression of Factors Associated with Moderate-to-Severe Generalized Anxiety Symptoms.

Variables	OR	(95% CI)	*p* Value
Age, years	0.97	(0.96, 0.98)	<0.001
Nationality, Saudi	0.95	(0.64, 1.41)	0.8
Health status, disease existence	1.58	(1.21, 2.06)	<0.001
Weight status, overweight and/or obesity	1.35	(1.07, 1.71)	0.009
Education, with a university degree	0.89	(0.70, 1.13)	0.35
High self-esteem	0.67	(0.53, 0.83)	<0.001
High quality of life	0.67	(0.54, 0.85)	<0.001
High health satisfaction	0.59	(0.47, 0.74)	<0.001
Daily intake of vegetables	0.88	(0.68, 1.15)	0.3
Daily intake of fruits	0.97	(0.68, 1.39)	0.8
Daily intake of snacks	1.29	(1.03, 1.61)	0.02
Daily intake of soft drinks	1.52	(1.09, 2.13)	0.01
Daily intake of breakfast	0.86	(0.69, 1.06)	0.1

Abbreviations: CI, confidence interval; n, number of complete cases; OR, odds ratio. The female-only analysis was based on complete cases (n = 1849).

## Data Availability

The data are not publicly available due to privacy and ethical considerations. De-identified data may be available from the corresponding author upon reasonable request and subject to institutional approval.
